# HOW MEDICAL EDUCATION ALLEVIATED ETHICAL DILEMMAS IN RESIDENTS DURING END-OF-LIFE FAMILY MEETINGS IN THE SOUTH TEXAS

**DOI:** 10.1093/geroni/igz038.1085

**Published:** 2019-11-08

**Authors:** Jessica G Martin, Andrya Rivera-Burciaga, Cesar Gutierrez, Andrew Dentino

**Affiliations:** 1 University of Texas Rio Grande Valley, Edinburg, Texas, United States; 2 DHR Health, Edinburg, Texas, United States

## Abstract

The UTRGV DHR Internal Medicine Program conducted a study addressing end of life (EOL) care focused on our Hispanic community in regards to communication and trust between patients, caregivers, and healthcare providers. Our residents train at a community hospital which cares for an 89% Hispanic population of 1.2 million, spanning over 4 counties of the Rio Grande Valley. Trainees are often involved in family meetings while treating hospitalized, terminally ill patients. Although family meetings are a standard approach in palliative care, Hispanic family meetings tend to occur more often and with a larger, extended family unit. Our intent was to educate our residents to initiate conversations about EOL care choices promoting delivery of patient-centered, family oriented care utilizing culturally appropriate information regarding EOL issues. Baseline surveys were provided to all 39 trainees which assessed anxiety, incompetence, and communication skills in delivering bad news during family meetings. An advanced care planning process was implemented over 3 months with a goal to engage patients in EOL conversations, initiation, and completion of advanced directives. Residents received weekly training on interactive methods and ethical concepts including group discussions, role-playing, and demonstrations which were culturally and linguistically appropriate. We found that physician competence in conducting Hispanic family meetings is vital. Residents completed a post-training survey resulting in 100% improved attitudes and behaviors such as confidence, satisfaction, caring and empathy. They felt more comfortable and prepared to speak to a larger family unit who was likely to ask a lot of questions and request multiple meetings.

